# Optimal medical therapy after percutaneous coronary intervention in very elderly patients with coronary artery disease^☆^

**DOI:** 10.1016/j.ijcrp.2022.200162

**Published:** 2022-11-30

**Authors:** Takamitsu Nakamura, Takeo Horikoshi, Tsuyoshi Kobayahi, Toru Yoshizaki, Manabu Uematsu, Yosuke Watanabe, Jun Nakamura, Aritaka Makino, Yukio Saito, Jun-ei Obata, Takao Sawanobori, Hajime Takano, Ken Umetani, Akinori Watanabe, Tetsuya Asakawa, Akira Sato

**Affiliations:** a Department of Cardiovascular Medicine, University of Yamanashi, Faculty of Medicine, Chuo, Japan; b Department of Cardiology, Kofu Municipal Hospital, Kofu, Japan; c Department of Cardiology, Fujieda Municipal General Hospital, Fujieda, Japan; d Department of Cardiology, Kofu Jonan Hospital, Kofu, Japan; e Department of Internal Medicine, Yamanashi Prefectural Central Hospital, Kofu, Japan; f Department of Cardiology, Yamanashi Kosei Hospital, Yamanashi, Japan

**Keywords:** Optimal medical therapy, Percutaneous coronary intervention, Elderly patients

## Abstract

Background: It is still unclear whether optimal medical therapy (OMT) after percutaneous coronary intervention (PCI) has beneficial effects on long-term clinical outcomes in patients aged ≥80 years with coronary artery disease (CAD). Methods: This study analyzed the time to the first major adverse clinical event including death or nonfatal myocardial infarction (MI), for up to 3 years after PCI using multicenter registry data. Data for 1056 patients aged > 80 years successfully treated with PCI were included in the analysis. OMT was defined as a combination of antiplatelet drug, statin, beta-blocker, and angiotensin-converting enzyme inhibitor/angiotensin II receptor blocker. Results: In total, 204 (19%) patients in this study received OMT and 852 (81%)　received sub-OMT. During a median follow-up of 725 days, adverse clinical events occurred in 183 patients (death, n=177; nonfatal MI, n=6). Kaplan-Meier analysis showed that patients who received OMT had a lower probability of adverse clinical events than those who received sub-OMT (p<0.01, log-rank test). Propensity score matching yielded 202 patient-pairs treated with OMT or sub-OMT, in whom 64 adverse clinical events (death, n=56, nonfatal MI, n=4) occurred during follow-up. OMT remained significant in the reduction of the risk of adverse clinical events in a multivariate Cox proportional hazards model (hazard ratio 0.44; 95% confidence interval 0.26–0.75; p=0.003). Conclusions: OMT after PCI was associated with significantly fewer adverse clinical events, including all-cause death and nonfatal MI, in patients aged ≥ 80 years with CAD. OMT might be safe and effective for these very elderly patients.

## 1 Introduction

Coronary artery disease (CAD) is one of the leading causes of mortality and morbidity in very elderly patients [1,2]. The advent of percutaneous coronary intervention (PCI) and technological improvements in PCI devices over recent decades have led to an increased number of very elderly patients with CAD being treated by PCI [3,4]. However, these patients have a higher prevalence of comorbidity and more complex lesions, leading to an increased risk of adverse clinical events after PCI [5]. Therefore, it is important to establish a therapeutic strategy that improves the clinical outcomes in these patients.

Each of the components of optimal medical therapy (OMT), namely, an antiplatelet agent, statin, beta-blocker, and angiotensin-converting enzyme inhibitor (ACE-I)/angiotensin II receptor blocker (ARB), has been associated with a significant reduction in the risk of cardiovascular events [[6], [7], [8]]. Moreover, recent clinical data have shown that OMT combined with revascularization therapy, including PCI or coronary artery bypass grafting (CABG), is beneficial regardless of the revascularization strategy used [9]. Therefore, evidence-based guidelines recommend use of OMT for patients with CAD after PCI [7]. However, most clinical guidelines for management of CAD have been developed based on the results of randomized controlled trials, in which elderly patients have often been excluded, even though they have the highest rate of medication use [10,11]. A recent observational study suggested that OMT has beneficial effects on long-term clinical outcomes in elderly patients with CAD [12,13]. However, it is still unclear whether these beneficial effects remain after balancing for selection bias in terms of clinical characteristics and established atherosclerotic risk factors.

Therefore, in this study, we sought to determine whether OMT is associated with a reduction in adverse clinical events, including all-cause death and nonfatal myocardial infarction (MI), among patients aged ≥80 years with CAD treated by PCI using propensity-score matched data from the Japanese multicenter observational PCI registry.

## 2 Methods

### 2.1 Study patients

The FUJISUN registry is a multicenter prospective observational registry containing data collected at the University of Yamanashi Hospital and 5 collaborating hospitals in Yamanashi and Shizuoka prefectures in Japan. This registry was designed to record clinical characteristics, PCI-related data, and outcomes after PCI. All prescribed medications and PCI procedures were at the discretion of the patients’ primary physicians. A data manager at each hospital is responsible for collecting clinical and PCI-related data. The FUJISUN registry contains data for 7173 patients who underwent PCI for CAD at any of the attending hospitals from May 2008 to December 2018. This registry is registered in the UMIN Clinical Trials Registry (unique identifier UMIN000047369). The protocol used to collect the FUJISUN data are approved by the ethics committees at all attending hospitals and performed in accordance with the principles outlined in the 1975 Declaration of Helsinki.

### 2.2 Study protocol

This study was a sub-analysis of the FUJISUN registry data. Patient data were analyzed according to whether OMT was administered (OMT group) or sub-OMT was provided (sub-OMT group) at discharge. OMT was defined as use of all four medications, namely, an antiplatelet agent, a statin, a beta-blocker, and an ACE-I/ARB. Sub-OMT was defined as omission of any of these four medications. We retrospectively investigated the time to the first major adverse clinical event for up to 3 years after enrollment. Adverse clinical events were defined as all-cause death and nonfatal MI. If the first hospitalization for MI culminated in death from progressive pump failure or sudden cardiac death during the follow-up period, the event was registered as death. Nonfatal MI was diagnosed by typical ischemic chest pain with a creatine kinase-MB level at least twice the upper limit of normal, a troponin T level >0.1 ng/ml, or characteristic ischemic changes on the electrocardiogram at the time of the event. Follow-up data were obtained by the patients’ primary physicians and collected by the data managers at each hospital. All endpoint data were strictly checked for accuracy, consistency, and completeness of follow-up by the investigators. Two of the investigators (T.N. and T.K.) checked all the data, carried out the analyses, and maintained the security of the data files. The need for written informed consent was waived because of the retrospective observational study design. Patients who died within 30 days after PCI and those receiving hemodialysis were excluded.

### 2.3 Statistical analysis

All descriptive data are expressed as the mean ± standard deviation or as the frequency (percentage). Mean values were compared between the two groups using the unpaired *t*-test and frequencies using the chi-squared test. Kaplan-Meier survival analyses were performed according to number of OMT medications used and whether the patients received OMT or sub-OMT. The ability of the clinical parameters and use of OMT were assessed using univariate and multivariate Cox proportional hazard models. The univariate Cox hazards analyses included age, male sex, history of MI, history of stroke, prior PCI, prior CABG, peripheral arterial disease (PAD), body mass index, New York Heart Association (NYHA) class II–IV, number of diseased vessels, left main coronary artery disease, left ventricular ejection fraction (LVEF) < 40%, current smoking, diabetes mellitus, hypertension, estimated glomerular filtration rate (eGFR), low-density lipoprotein (LDL) cholesterol, high-density lipoprotein (HDL) cholesterol, HbA_1c_, ST-elevation myocardial infarction (STEMI), and use of OMT. Variables with *p*-values <0.10 were selected in a stepwise multivariate Cox proportional hazards analysis with backward elimination. The hazard ratio (HR) and 95% confidence interval (CI) were estimated by 10 years for age, 10 mg/dL for LDL cholesterol and HDL cholesterol, and 10 ml/min/1.73 m^2^ for eGFR. Dichotomous variables were coded 1 for the presence of the factor and 0 for its absence. All probability values presented are two-tailed, with statistical significance being inferred at *p* < 0.05. The use of OMT were at the discretion of the primary physicians. Therefore, we calculated propensity scores to reduce selection bias with regard to patients treated with OMT. The propensity scores for each patient were calculated from a logistic regression model to predict the probability of being treated with OMT. In this logistic model, 20 covariates (age, male sex, history of MI, history of stroke, prior PCI, prior CABG, PAD, body mass index, NYHA class II–IV, number of diseased vessels, left main coronary artery disease, LVEF <40%, current smoking, diabetes mellitus, hypertension, eGFR, LDL-cholesterol, HDL-cholesterol, HbA_1c_, and STEMI) were used to calculate the propensity score. We matched patients using the nearest neighbor method with a 1:1 matching procedure without replacement and a caliper width of 0.05, calculated by 0.2 × standard deviation of the logit of the propensity score. Most of the statistical analyses were performed using STATA 16.0 software (StataCorp, College Station, TX, USA).

## 3 Results

### 3.1 Study patients

**Fig. 1:**
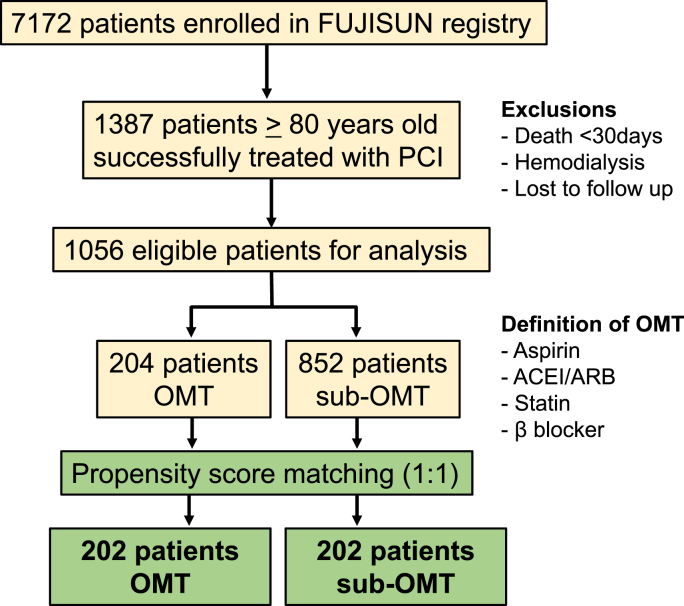
Flow of patients through the study.

This study initially included 1387 patients aged ≥80 years with CAD who were successfully treated with PCI during the study period. Based on our exclusion criteria, data for 96 patients who died within 30 days after PCI and 42 who received hemodialysis were excluded. Data for a further 193 patients who had been lost to follow-up were also excluded. Data for the remaining 1056 patients were included in the analysis. Two hundred and four (19%) of these patients received OMT and 852 (81%) received sub-OMT (Fig. 1). The baseline clinical characteristics of the study participants treated with OMT and sub-OMT before propensity score-matched analysis are shown in Table 1.

**Table 1:** Comparisons of clinical characteristics between patients treated with OMT and sub-OMT.

	All patients N = 1056			Propensity score matched patients N = 404		
	OMT N = 204	sub-OMT N = 852	P-value	OMT N = 202	sub-OMT N = 202	P-value
Age (yrs)	83 ± 3	84 ± 4	0.002	83 ± 3	83 ± 3	0.54
Gender, male (%)	128 (62.8)	521 (61.2)	0.67	127 (62.9)	119 (58.9)	0.42
History of MI	27 (13.2)	101 (11.9)	0.59	27 (13.4)	24 (11.9)	0.65
History of stroke	26 (12.8)	90 (10.6)	0.37	26 (12.9)	23 (11.4)	0.65
Prior PCI	33 (16.2)	105 (12.3)	0.14	33 (16.3)	31 (15.4)	0.79
Prior CABG	7 (3.4)	23 (2.7)	0.57	7 (3.5)	12 (5.9)	0.24
PAD	12 (5.9)	53 (6.2)	0.86	12 (5.9)	10 (5.0)	0.66
BMI (kg/m^2^)	22.3 ± 2.9	22.2 ± 3.5	0.62	22.3 ± 2.9	22.4 ± 3.3	0.67
NYHA II-IV	94 (46.1)	395 (46.4)	0.94	93 (46.0)	93 (46.0)	1.00
Number of diseased vessels	1.7 ± 0.7	1.7 ± 0.8	0.34	1.7 ± 0.7	1.8 ± 0.8	0.85
LMCA disease	14 (6.9)	46 (5.4)	0.42	14 (6.9)	16 (7.9)	0.70
LVEF <40%	30 (14.7)	123 (14.4)	0.92	30 (14.9)	29 (14.4)	0.89
Current smoking	26 (12.8)	89 (10.5)	0.34	26 (12.9)	29 (14.4)	0.66
Diabetes mellitus	100 (49.0)	305 (35.8)	<0.001	98 (48.5)	98 (48.5)	1.00
Hypertension	175 (85.8)	635 (74.5)	0.001	173 (85.6)	170 (84.2)	0.68
eGFR (ml/min/1.73m^2^)	52.9 ± 17.9	55.2 ± 16.9	0.16	52.8 ± 17.9	52.2 ± 16.5	0.72
LDL-cholesterol (mg/dl)	108 ± 37.7	104 ± 32.4	0.13	108 ± 37.8	109 ± 33.9	0.77
HDL-cholesterol (mg/dl)	47.9 ± 13.3	49.9 ± 15.9	0.09	48.0 ± 13.2	47.2 ± 14.1	0.54
HbA1c (%)	6.3 ± 1.1	6.0 ± 1.1	0.0001	6.2 ± 0.9	6.2 ± 1.1	0.38
STEMI, n (%)	90 (44.1)	336 (39.4)	0.22	89 (44.1)	93 (46.0)	0.69
OMT use, n (%)						
Aspirin	204 (100)	839 (98.5)	0.08	202 (100)	200 (99.0)	0.16
ACEI/ARB	204 (100)	446 (52.4)	<0.0001	202 (100)	116 (57.4)	<0.0001
Beta-blocker	204 (100)	224 (26.3)	<0.0001	202 (100)	52 (25.7)	<0.0001
Statin	204 (100)	508 (59.6)	<0.0001	202 (100)	128 (63.4)	<0.0001

Data are expressed as mean ± SD or number (%) of patients.

Diabetes mellitus was defined according to the American Diabetes Association criteria or use of antidiabetic medications.

Hypertension was defined as >140/90 mmHg or use of antihypertensive medication.

Abbreviations: MI: myocardial infarction, PCI: percutaneous coronary intervention, CABG: coronary artery bypass grafting, PAD: peripheral artery disease, BMI: body mass index, LMCA: left main coronary artery, LVEF: left ventricular ejection fraction, eGFR: estimated glomerular filtration rate, LDL: low density lipoprotein, HDL: high density lipoprotein, STEMI: ST-elevation myocardial infarction, ACEI: angiotensin converting enzyme inhibitor, ARB: angiotensin II receptor blocker.

### 3.2 Clinical characteristics in the OMT and sub-OMT groups

The baseline clinical characteristics are compared in Table 1 according to whether OMT or sub-OMT was provided. Before propensity score matching, patients in the OMT group had significantly higher frequencies of diabetes mellitus and hypertension, higher HbA1c levels, and were significantly younger than those in the sub-OMT group (Table 1). Patients in the OMT group also had significantly higher rates of ACE/ARB, beta-blocker, and statin use than those in the sub-OMT group (Table 1).

### 3.3 Clinical outcomes

**Fig. 2:**
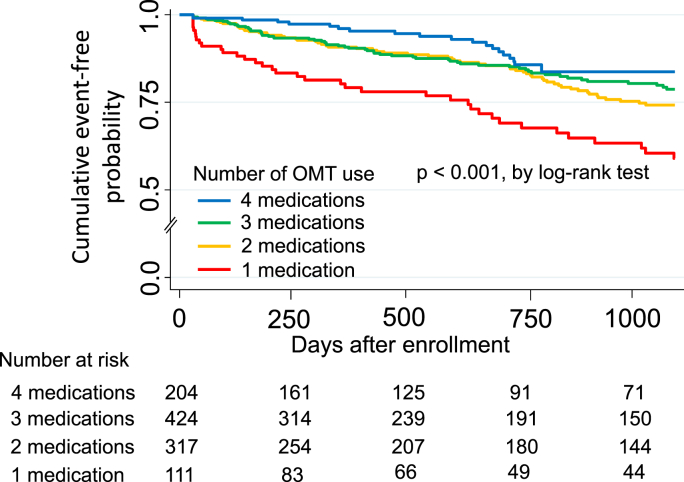
Kaplan-Meier curves showing event-free probability of adverse clinical events in all study patients.

**Fig. 3:**
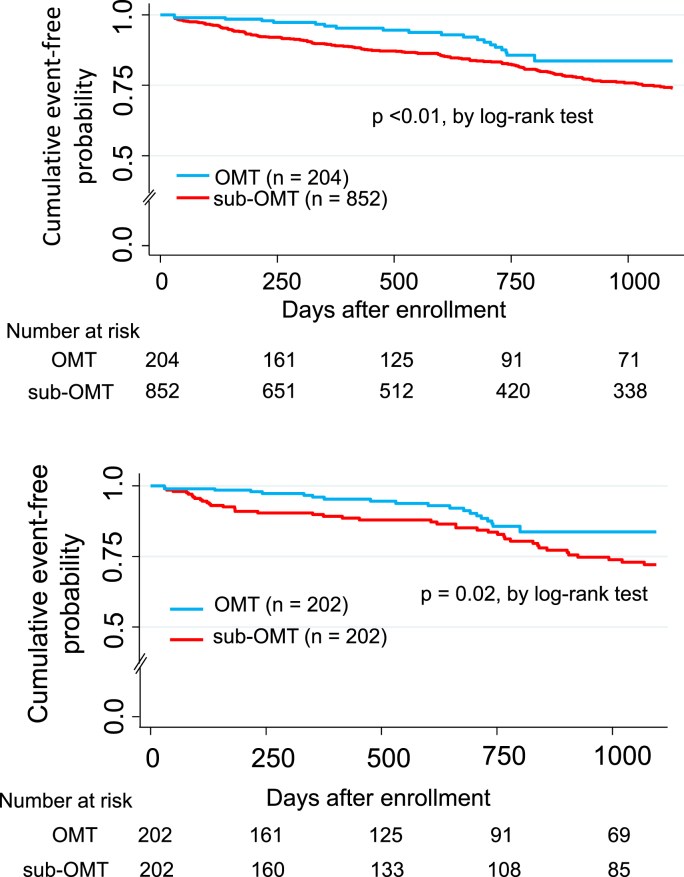
Kaplan-Meier curves showing event-free probability of adverse clinical events according to whether patients received OMT or sub-OMT (upper panel). Kaplan-Meier curves showing event-free probability of adverse clinical events in propensity score-matched patients (lower panel).

Adverse clinical events occurred in 183 patients (death, n = 177; nonfatal MI, n = 6) during 30–1095 days of follow-up (median 725 days [interquartile range, 266–1095]). Kaplan-Meier analysis showed that the probability of adverse clinical events was significantly lower in patients treated with OMT than in those treated with three, two, or one of the components of OMT (p < 0.001, log-rank test; Fig. 2). Moreover, when the clinical adverse clinical outcomes were stratified according to whether OMT or sub-OMT was used, Kaplan-Meier analysis showed that the adverse clinical event rate over time patients was significantly lower in the OMT group than in the sub-OMT group (p < 0.01, log-rank test; upper panel Fig. 3).

### 3.4 Propensity score-matched analysis

Propensity score matching resulted in data for 202 patient-pairs treated with OMT or sub-OMT for analysis (Fig. 1). There was no significant between-group difference in the distribution of clinical characteristics after propensity score matching, as shown in Table 1. A total of 64 adverse clinical events (death, n = 59; nonfatal MI, n = 5) occurred in the propensity score-matched patients during follow-up. The incidence of adverse clinical events was significantly lower in patients treated with OMT than in those treated with sub-OMT (10.3% [n = 21] vs. 21.1% [n = 43]; p < 0.01). Patients with adverse clinical events had a significantly greater number of diseased vessels, a lower HDL cholesterol level, were more likely to have a history of MI, prior PCI, PAD, NYHA class II–IV disease, an LVEF <40%, to be current smokers, and were less likely to receive OMT, an ACE/ARB, or a statin (Table 2).

**Table 2:** Comparisons of clinical characteristics between patients with and without adverse clinical events using propensity score matched patients.

	With events N = 60	Without events N = 344	P-value
Age (yrs)	84 ± 3	83 ± 3	0.11
Gender, male (%)	38 (63.3)	208 (60.5)	0.67
History of MI	14 (23.3)	37 (10.8)	0.007
History of stroke	7 (11.7)	42 (12.2)	0.91
Prior PCI	15 (25.0)	49 (14.2)	0.04
Prior CABG	5 (8.3)	14 (4.1)	0.15
PAD	8 (13.3)	14 (4.1)	0.004
BMI (kg/m^2^)	22.0 ± 3.4	22.4 ± 3.1	0.33
NYHA II-IV	36 (60.0)	150 (43.6)	0.02
Number of diseased vessels	1.9 ± 0.8	1.7 ± 0.7	<0.05
LMCA disease	7 (11.7)	23 (6.7)	0.18
LVEF <40%	17 (28.3)	42 (12.2)	0.001
Current smoking	13 (21.7)	42 (12.2)	<0.05
Diabetes mellitus	35 (58.3)	161 (46.8)	0.10
Hypertension	50 (83.3)	293 (85.2)	0.71
eGFR (ml/min/1.73m^2^)	49.1 ± 16.5	53.0 ± 17.2	0.10
LDL-cholesterol (mg/dl)	103 ± 33.1	109 ± 36.2	0.16
HDL-cholesterol (mg/dl)	42 ± 13.7	48 ± 13.4	0.0002
HbA1c (%)	6.2 ± 1.4	6.2 ± 0.9	0.94
STEMI, n (%)	31 (51.7)	151 (43.9)	0.26
Medication use, n (%)			
OMT	21 (35.0)	181 (52.6)	0.01
Aspirin	60 (100)	342 (99.4)	0.55
ACE/ARB	40 (66.7)	278 (80.8)	0.01
Statin	37 (61.7)	293 (85.2)	<0.001
Beta-blocker	29 (48.3)	225 (65.4)	0.01

Abbreviations as in Table 1.

Kaplan-Meier analysis also showed that the probability of adverse clinical events was lower in the OMT group (n = 202) than in the sub-OMT group (n = 202), which is similar to the results obtained for all the study patients (p = 0.02, log-rank test; Fig. 3, lower panel). As shown in Table 3, univariate Cox proportional hazard analysis showed that age (HR 2.60; 95% CI 1.26–5.36; p = 0.01), PAD (HR 3.61; 95% CI 1.71–7.62; p = 0.001), NYHA class II–IV disease (HR 2.11; 95% CI 1.26–3.54; p = 0.005), number of diseased vessels (HR 1.40; 95% CI 1.02–1.94; p = 0.04), LVEF <40% (HR 2.60; 95% CI 1.48–4.56; p = 0.001), HDL cholesterol (HR 0.66; 95% CI 0.53–0.82; p = 0.001), and OMT (HR 0.54; 95% CI 0.32–0.91; p = 0.02) (Table 3). Moreover, age (HR 3.00; 95% CI 1.51–5.96; p = 0.002), history of MI (HR 2.83; 95% CI 1.53–5.24; p = 0.001)), LVEF <40% (HR 2.02; 95% CI 1.11–3.70; p = 0.02), current smoking (HR 2.26; 95% CI 1.19–4.30; p = 0.01), HDL cholesterol (HR 0.67; 95% CI 0.54–0.84; p = 0.001), and OMT (HR 0.44; 95% CI 0.26–0.75; p = 0.003) remained significant in the multivariate Cox proportional hazard analysis. In multivariate Cox proportional hazards analysis, the risk of adverse clinical events was reduced significantly by 56% in patients treated with OMT (Table 3).

**Table 3:** Univariate and multivariate Cox hazard analysis for adverse clinical events using propensity matched patients.

	Univariate analysis			Multivariate analysis		
	HR	95% CI	P-value	HR	95% CI	P-value
Age (yrs)	2.60	1.26–5.36	0.01	3.00	1.51–5.96	0.002
Gender, male	1.15	0.68–1.94	0.61		Not selected	
History of MI	2.15	1.18–3.91	0.01	2.83	1.53–5.24	0.001
History of stroke	0.84	0.38–1.84	0.66		Not selected	
Prior PCI	1.67	0.93–2.99	0.09		Not selected	
Prior CABG	1.46	0.58–3.66	0.42		Not selected	
PAD	3.61	1.71–7.62	0.001		Not selected	
BMI (kg/m^2^)	0.96	0.88–1.04	0.28		Not selected	
NYHA II-IV	2.11	1.26–3.54	0.005	1.67	0.97–2.88	0.07
Number of diseased vessels	1.40	1.02–1.94	0.04		Not selected	
LMCA disease	1.89	0.86–4.15	0.12		Not selected	
LVEF <40%	2.60	1.48–4.56	0.001	2.02	1.11–3.70	0.02
Current smoking	1.77	0.96–3.27	0.07	2.26	1.19–4.30	0.01
Diabetes mellitus	1.51	0.90–2.52	0.12		Not selected	
Hypertension	0.92	0.47–1.82	0.82		Not selected	
eGFR (ml/min/1.73m^2^)	0.87	0.74–1.01	0.07		Not selected	
LDL-cholesterol (mg/dl)	0.94	0.86–1.01	0.10		Not selected	
HDL-cholesterol (mg/dl)	0.66	0.53–0.82	0.001	0.67	0.54–0.84	0.001
HbA1c (%)	1.01	0.78–1.32	0.91		Not selected	
STEMI, n (%)	1.42	0.85–2.35	0.18		Not selected	
OMT	0.54	0.32–0.91	0.02	0.44	0.26–0.75	0.003

HR: hazard ratio, CI: confidence interval, other abbreviations as in Table 1.

The HR and 95% CI were estimated by 10 years for age, 10 mg/dL for LDL-cholesterol, and HDL-cholesterol, and 10 ml/min/1.73 m^2^ for eGFR.

## 4 Discussion

In this multicenter observational study, OMT, including aspirin, an ACEI/ARB, a statin, and a beta-blocker, was used in only 19% of patients aged ≥80 years after PCI for CAD. However, use of OMT in these patients was associated with a significant reduction in the risk of adverse clinical events after PCI, including all-cause death and nonfatal MI. Moreover, the beneficial effects of OMT on the risk of adverse clinical events remained significant in the propensity score-matched data. Therefore, OMT might be safe and effective for well selected patients aged ≥80 years with CAD treated by PCI.

Use of the individual components of OMT have been associated with a reduction in the risk of cardiovascular events in patients with CAD, whether they were treated conservatively or with revascularization [9,14,15]. Therefore, recent clinical guidelines for treatment of CAD recommend use of evidence-based therapies for all eligible [7,16]. However, although the benefits of OMT for secondary prevention in elderly patients have been recognized, treatment gaps have been identified in real-world practice that suggest adherence to recommendations is suboptimal in elderly patients [17]. These results are consistent with our findings that patients who received OMT were older than those who received sub-OMT and that only 19% of patients aged ≥80 years with CAD received OMT after PCI. Moreover, in our study, patients who received OMT were significantly younger than those who received sub-OMT. There are several potential age-related non-cardiac explanations for these treatment gaps, including drug-drug interactions, polypharmacy, and inappropriate medications that might result in life-threatening renal dysfunction [11,18] and make clinicians less willing to use OMT in elderly patients after PCI.

In this study, use of OMT significantly reduced the risk of death and nonfatal MI, indicating that all components of OMT are important for reducing adverse clinical events. Recent clinical guidelines recommend use of an antiplatelet agent in all patients with CAD who have no contraindications [7,16]. The finding that antiplatelet therapy was used after PCI in 98% of our study participants is consistent with this recommendation. However, continuous use of antiplatelet therapy in elderly patients can be challenging because they are more prone to bleeding complications than their younger counterparts [6]. The high rate of use of aspirin in our study may reflect our inclusion criteria, whereby only patients treated with PCI were included, and suggests that most patients were considered able to tolerate antiplatelet therapy. Statin therapy is also recommended for secondary prevention of CAD in younger patients [7,16]. However, whether using a statin for secondary prevention of CAD in patients ≥80 years of age has an advantage remains controversial. Therefore, evaluation of adverse effects and drug-drug interactions before using a statin in patients ≥75 years of age has been recommended [7]. We have recently demonstrated that statin treatment was effective for reducing adverse clinical events after PCI in patients with CAD and aged ≥75 years [19]. In this sense, our present results support our previous finding that statins may be effective in older patients with CAD after PCI if there are no contraindications. An ACE-I/ARB and a beta-blocker are also strongly recommended for patients with CAD, hypertension, diabetes mellitus, and heart failure [7]. However, in our present study, we found that an ACE-I/ARB and a beta-blocker were used less often than aspirin and statins. Recent clinical data have shown that use of an ACE-I or ARB increases the risk of renal dysfunction, vascular edema, and hypotension and that use of a beta-blocker increases the risk of hypotension and bradycardia in elderly patients despite the beneficial effects of these drugs [18]. Therefore, the potential risk of adverse effects might be one of the reasons for underuse of ACE-Is/ARBs and beta-blockers in elderly patients with CAD.

A recent single-center observational study suggested that OMT has beneficial effects on clinical outcomes in elderly patients aged >80 years with acute coronary syndrome [13], which is also consistent with our findings. However, our present multicenter observational study also found that the beneficial effects of OMT extended to a reduction of adverse clinical outcomes after PCI, which persisted after propensity score matching to balance the clinical factors known to influence the decision to use OMT. Therefore, the findings of this study suggest that patients aged ≥80 years with CAD and no contraindications are likely to derive benefit from OMT after PCI.

## 5 Limitations

This study has several limitations. First, it included a relatively small number of patients with CAD, which reduced the statistical power of the study. Second, we did not collect data on adherence with medication during follow-up, which might have affected clinical outcomes. Therefore, larger clinical trials that include follow-up data on medication are needed to assess the precise role of adherence with OMT in the prognosis of elderly patients with CAD. Third, we did not collect information on use of OMT before PCI. Moreover, we only included patients without adverse clinical events in the first 30 days post-PCI, and use of OMT before PCI might have affected the clinical outcomes. Third, although propensity score analysis allowed us to balance the data for the two groups using variables that are collected by this multicenter registry, there might have been some degree of selection bias stemming from other variables that could not observed in the present study. Fourth, we could not examine the duration and the effects of dual-antiplatelet therapy on clinical outcomes in the present study. It may be possible that the favorable clinical outcomes of OMT was due to the effects of medication including dual-antiplatelet therapy. Therefore, a larger clinical trial is needed to examine the effect of OMT including dual-antiplatelet therapy on clinical outcomes in CAD patients >80 years old. Finally, we focused only on use of OMT but not consider achievement of optimal goals for risk factors. Therefore, the patients treated with OMT may not have been optimized in terms of achievement of goals for risk factors. A larger prospective trial that uses the same doses of OMT with achievement of the risk factor goals is needed to confirm whether OMT might be effective for reducing adverse clinical outcomes in elderly CAD patients after PCI.

## 6 Conclusions

In this study, use of OMT in patients aged ≥80 years with CAD was associated with a significant reduction in the risk of adverse clinical events, including all-cause death and nonfatal MI after PCI. Therefore, OMT might be safe and effective for selected patients aged ≥80 years with CAD after PCI.

## Declaration of conflict of interest

None.

## Sources of funding

This study was supported by a Grant-in-Aid for Scientific Research (21K07387) from the Japan Society for the Promotion of Science, Japan.

## Credit author statement

**Takamitsu Nakamura**: Conceptualization, Methodology, Writing- Original draft preparation. **Takeo Horikoshi**: Data analysis, Writing- Original draft preparation. **Tsuyoshi Kobayashi**: Investigation, **Toru Yoshizaki**: Investigation, Data curation, **Manabu Uematsu**: Investigation, Writing- Reviewing and Editing, **Yosuke Watanabe**: Investigation, Writing- Reviewing and Editing, **Jun Nakamura**: Investigation, **Aritaka Makino**: Investigation, **Yukio Saito**: Investigation, **Jun-ei Obata**: Investigation, **Takao Sawanobori**: Investigation, **Hajime Takano**: Investigation, **Ken Umetani**: Investigation, **Akinori Watanabe**: Investigation, **Tetsuya Asakawa**: Investigation, **Akira Sato**: Supervision.
